# Six-Year Single-Center Experience with ECMO Use in Various Strategies for Lung Transplantation, Including COVID-19 Patients

**DOI:** 10.3390/jcm14124195

**Published:** 2025-06-12

**Authors:** Tomasz Stącel, Kamil Kegler, Paweł Sybila, Agata Mędrala, Małgorzata Jekiełek, Mirosław Nęcki, Piotr Pasek, Anna Pióro-Lewandowska, Piotr Przybyłowski, Maciej Urlik

**Affiliations:** 1 Department of Lung Transplantation, Silesian Centre for Heart Diseases in Zabrze, 41-800 Zabrze, Poland; 2 5th Military Clinical Hospital in Krakow, 30-901 Krakow, Poland; 3 1st Department of Cardiology, Silesian Centre for Heart Diseases in Zabrze, 41-800 Zabrze, Poland; 4 Regional Specialised Hospital No. 4 in Bytom, 41-902 Bytom, Poland; 5 Department of Physiotherapy, Faculty of Health Sciences, Jagiellonian University Collegium Medicum, 31-008 Krakow, Poland; 6 Department of Interstitial Lung Diseases and Transplantology, the Saint Paul II Hospital, 31-202 Krakow, Poland; 7 Centre for Transplantology and Interstitial Lung Diseases, Jagiellonian University, 31-202 Krakow, Poland; 8 Department of Cardiac Anaesthesia and Intensive Care, Silesian Centre for Heart Diseases in Zabrze, 41-800 Zabrze, Poland; 9 Faculty of Medical Sciences in Zabrze, Medical University of Silesia, 40-055 Katowice, Poland; 10 Silesian Centre for Heart Diseases in Zabrze, 41-800 Zabrze, Poland

**Keywords:** ECMO, lung transplantation, COVID-19 transplantation, awake-ECMO, ECMO-bridge, sport-ECMO, left ventricular conditioning

## Abstract

**Objectives:** This study aims to describe the experience of using ECMO on various patients who require ECMO support during the entire perioperative period of lung transplantation. ECMO has several roles: it can bridge patients to transplantation, improve lung graft function in case of primary graft dysfunction after transplantation, improve left ventricle function after transplantation in patients with primary pulmonary hypertension, and manage COVID-19 patients who are awaiting LuTx or undergoing LTx. **Methods:** We present 6-year results from a high-volume lung transplant center (219 cases/6 years, >50 cases/2022). We used ECMO in 56 cases (25.6%) of all lung transplants between 2018 and 2023. **Results:** The one-year survival rate of patients transplanted on ECMO was 85.7%. We applied all advanced ECMO techniques, such as bridging to transplantation on ECMO (*n* = 15, early survival 66.7%) and left ventricular conditioning after LuTx with VA-ECMO (*n* = 12, 60-day and one-year survival 85.1% and 53%, respectively). We also bridged patients with COVID-19 to transplantation and transplanted them from ECMO (*n* = 9, early survival 55%). **Conclusions:** This article shows possible applications of ECMO therapy for various indications in lung transplant patients and, along with data from other publications, it demonstrates that ECMO can improve survival and outcomes for patients with respiratory failure, primary pulmonary hypertension, and COVID-19. The COVID-19 pandemic highlighted new utilization of ECMO, demonstrating its usefulness and importance in critical care medicine. Further research into capabilities of the ECMO system may expand the knowledge about its role in lung transplantation and future treatments.

## 1. Introduction

Lung transplantation is the definitive treatment for patients with end-stage lung disease who have utilized all other medical and surgical options [1]. Lung transplantation is usually performed without extracorporeal support, but sometimes conventional cardiopulmonary bypass or extracorporeal membrane oxygenation (ECMO) is required during the surgery [2,3].

ECMO is a technique that can support or temporarily replace the function of the lungs and/or the heart in patients with respiratory or cardiac failure. ECMO can also be used as a bridge to lung transplantation, meaning that it can help patients who are waiting for a suitable donor organ to survive and improve their condition [4,5]. This is especially important for patients who are critically ill and have a high risk of dying on the waiting list.

**Figure 1:**
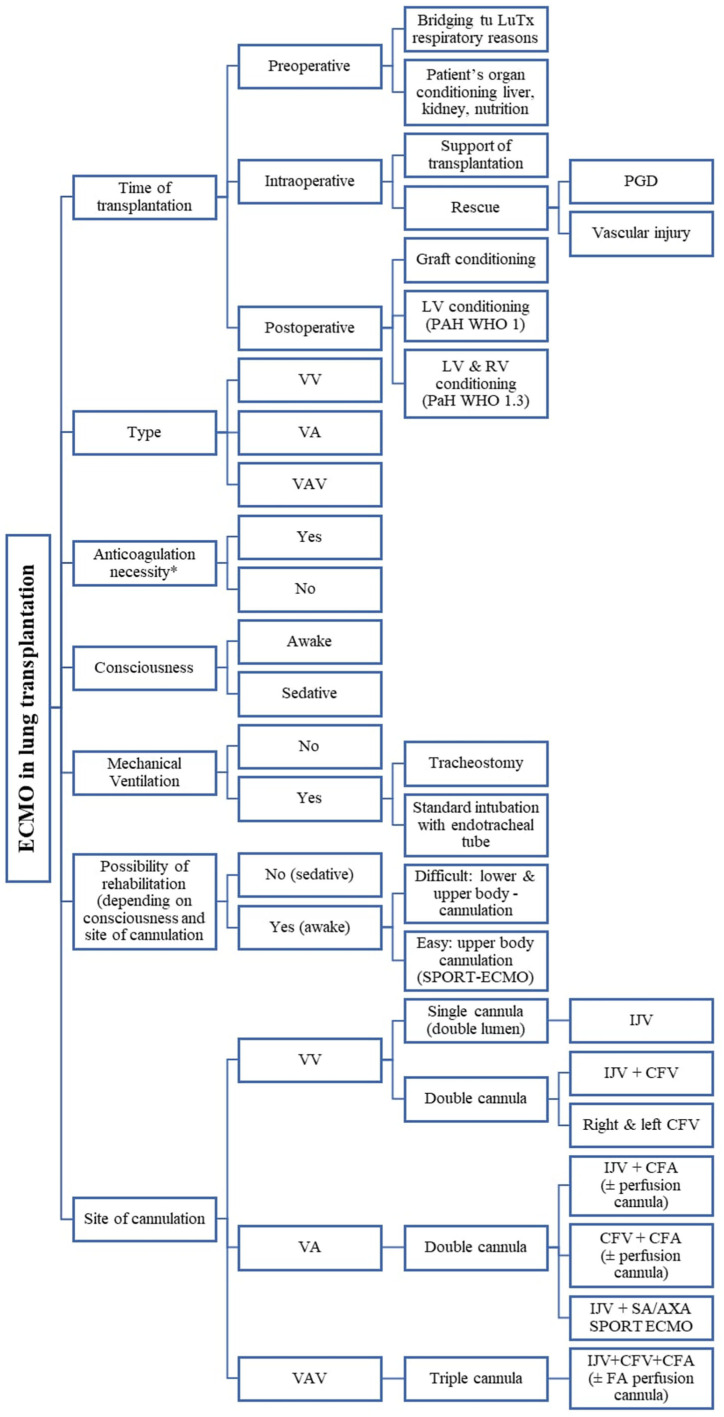
Illustration of various types of ECMO applications, configurations, and nomenclature depending on cannulation topography, patient’s level of consciousness, and other clinical factors. VV—veno-venous; VA—veno-arterial; VAV—veno-arterio-venous; PGD—primary graft dysfunction; LV—left ventricle; RV—right ventricle; PAH—pulmonary artery hypertension; IJV—internal jugular vein; CFV—common femoral vein; CFA—common femoral vein; FA—femoral artery; LuTx—lung transplantation; SA—subclavian artery; AXA—axillary artery.

There are many types of ECMO strategies and techniques as well as combinations between them, depending on the clinical situation and the goals of therapy in the lung transplantation process [6] (Figure 1). These include different modes of ECMO: veno-venous (VV), veno-arterial (VA), veno-arterio-venous (VAV), different cannulation sites (central, peripheral, single-site, dual-site), different levels of support (partial/full), depending on patient consciousness level: awake or sedative (according to Richmond Agitation–Sedation Scale (RASS)), and stationary or transport ECMO.

It is essential to have a thorough knowledge of these ECMO options, as well as their indications and contraindications, in order to achieve the best possible outcomes for lung transplant candidates and recipients. ECMO has also become a lifesaving tool in the era of COVID-19, as it can provide a bridge to recovery or bridge to transplantation for patients with severe acute respiratory distress syndrome caused by the novel coronavirus SARS-CoV-2 (CARDS in opposite to ARDS) [7]. However, ECMO still requires further development and improvement in terms of transportability, miniaturization, biocompatibility, anticoagulation, and complication management [8]. Nowadays, ECMO therapy is becoming a very individualized therapy due to the choice of the optimal moment of its implementation, very diverse indications for its use and, as the COVID-19 pandemic has shown, its ever-expanding applications. Therefore, when using this technique, it is worth taking all these factors into account. Our study is an attempt to provide insight into the applications of this device, technique, and its role in lung transplantation. We share the results achieved in the authors’ center against the background of the results of other European and world centers.

## 2. Materials and Method

The authors of this article present a 6-year experience. We share outcomes as well as our insights and challenges in applying different ECMO strategies and techniques. This study included all patients from our center who received ECMO (both bridging, intraoperative, and postoperative).

During the analysis of the results, possible ECMO modes were described, including AWAKE ECMO (all and divided into intubated and extubated) and SPORT ECMO. In these three modes, patients were not given neuromuscular blocking agents. Patients who received these ECMO modes had a score of 0 using The Richmond Agitation Sedation Scale assessing the state of consciousness. The inclusion criteria for the bridging group were: no possibility of further mechanical ventilation support, deteriorating hemodynamic condition, non-responsive to pharmacological treatment. In addition, the inclusion criteria for the study included all lung transplant patients with significant pulmonary hypertension, patients with lung damage caused by COVID-19, patients undergoing retransplantation, and patients with postoperative graft or left ventricular dysfunction.

Possible adverse events associated with therapy were: death during the ECMO procedure, neurological complications (stroke, impaired consciousness, peroneal nerve palsy), hemorrhagic (bleeding), and complications related to cannulation (limb ischemia, bleeding around the cannula, swelling or infection of the cannula implantation site). Possible adverse events of heparin use or non-use were death during ECMO procedure, neurological complications (stroke, impaired consciousness), and hemorrhagic complications (bleeding around the cannula, bleeding into the pleural cavity).

In our study, we use the concept of Left Ventricular Conditioning (LV conditioning), which involves intraoperative and postoperative use of VA ECMO in patients with pulmonary arterial hypertension who required lung transplantation. The consequence of pulmonary arterial hypertension is the loss of left ventricular mass, probably due to reduced left heart preload. After lung transplantation and normalization of pulmonary artery pressure, pulmonary edema may occur as a result of reduced left ventricular capacity. For this reason, the use of VA ECMO mode may support the work of the left ventricle [9,10,11,12].

The Ethics Committee of Medical University of Silesia reviewed the study protocol and confirmed that formal ethical approval was not required BNW/NWN/0052/KB/209/23.

Data are presented as mean ±SD for normally distributed quantitative variables and median with IQR for non-normally distributed ones. Survival analysis in patients receiving ECMO was performed using the Kaplan–Meier method with log-rank test to compare curves. Linear regression analysis was used to calculate trends and variability in the number of procedures (transplantations) over time. *p* values <0.05 were considered significant. Analyses were performed using the R programming language iRStudio environment (RStudio Team (2023). RStudio: Integrated Development for R. RStudio, Inc., Boston, MA, USA).

## 3. Results

### 3.1. Key Outcomes

Over six years (2018–2023) we performed 219 lung transplantations (double lung transplantations (DLTx) accounted for 97.3%; n = 213) and ECMO was used in 56 cases of them (25.6%) in almost all types of pulmonary diseases, both in adults and children (n = 9; 4.1%), including rare diseases and lung retransplantations in the late period (years) after primary LuTx for various indications, mainly due to chronic lung allograft dysfunction (CLAD) (Table 1). The characteristics of the patients and ECMO runs are presented in Table 2 and Table 3.

A summary of procedures, strategies, techniques, and modes of conducting all the patients on ECMO in our department in the period before lung transplantation, during transplantation, and after transplantation is presented in Table 4.

In each year, the number of LuTx performed with ECMO support increased, and the trend of that increase was statistically significant (*p* = 0.03) in our material (Table 4).

This increase was noticeable since 2020, when a new disease entity—COVID-19—appeared, and the experience gained then allowed people to still use ECMO, especially in patients with pulmonary hypertension (PH). The number of ECMO procedures performed in patients with iPAH is presented in Table 4.

Another example of the increase in the frequency of ECMO applications in lung transplantations is the fact that in 2023 alone, out of 36 LuTx, the ECMO system was used 12 times, which is 33.3% of the total number of transplants that year (Table 4).

The number of bridges to transplantation (BTT) was 15, which is 25.0% of all ECMO runs performed in our center (Table 4). BTT patients had a statistically significantly longer ECMO duration (*p* < 0.001), a more frequent need for mechanical ventilation before ECMO (*p* < 0.001), and more frequent renal and neurological complications prior to ECMO (Table 5).

Among patients bridged to transplantation with ECMO (*n* = 15), 9 (60.0%) had post-COVID-19 pulmonary fibrosis as the indication for lung transplantation. This subgroup constituted 16.1% of all LuTx patients on ECMO and 4.1% of the total transplant population (*n* = 219) (Table 4). All COVID-19-related cases were managed with VV-ECMO only.

**Figure 2:**
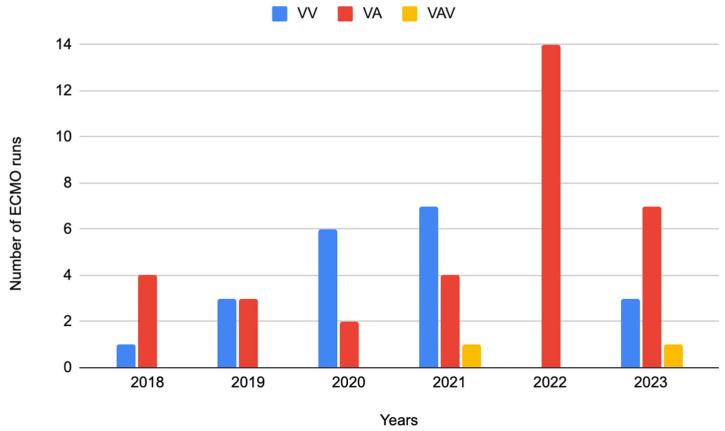
ECMO modes in individual years: VV, VA, and VAV performed in the years 2018–2023, VV—veno-venous, VA—veno-arterial, VAV—veno-arterial-venous.

The leading mode of ECMO was VA-ECMO (*n* = 34, i.e., 60.7%), followed by VV- ECMO (*n* = 20, i.e., 35.7%), and in a much smaller amount, VAV (*n* = 2, i.e., 3.6%) (Figure 2).

Of the 20 patients supported by VV-ECMO, 9 (45.0%) had COVID-19-related lung injury and were considered for lung transplantation (i.e., COVID-BTT). These patients had significantly longer ECMO duration (median 456 h). In contrast, none of the VA-ECMO patients were treated for COVID-19-related disease, and most received intraoperative or postoperative ECMO for hemodynamic support (Table 6).

The median total time that patients spent on ECMO support in our population was 66.2 h (Table 3). It can be assumed that the time a given patient was supported by the ECMO system is a surrogate for the severity of the patient’s clinical condition at the time of ECMO implantation.

The mortality rate in patients undergoing lung transplantation with ECMO therapy (regardless of whether before or after 30 days of transplantation) and the 30-day mortality rate after transplantation in this group of patients were 17.9% and 12.5%, respectively (Table 7). The most frequent complications in lung transplant patients treated with ECMO were hemorrhagic (21.4%), neurological (8.9%), and cannulation site complications (5.4%).

**Figure 3:**
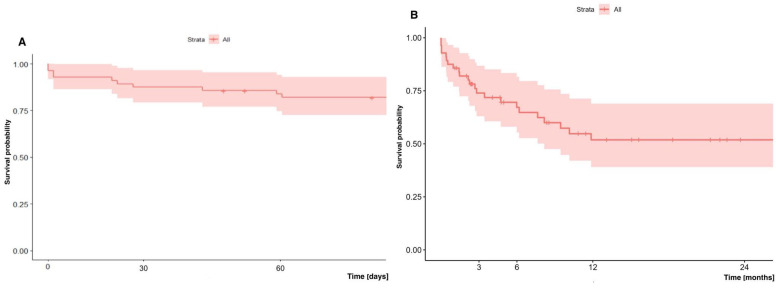
(**A**) Early (60-day) and (**B**) long-term (two-year) survival of patients undergoing lung transplantation with ECMO.

The early (60-day) and long-term (two-year) survival of patients who underwent lung transplantation with ECMO support (regardless of the type: VV vs. VA, cannulation method, consciousness state) was 82.0% and 51.9%, respectively (Figure 3). The median hospital stay after ECMO decannulation was 29 days (Table 3).

### 3.2. VV-ECMO vs. VA-ECMO—Outcomes

We performed a comparative analysis of patients’ survival depending on which form of ECMO therapy was used: VV (*n* = 20/56; 35.7%) vs. VA (*n* = 36/56; 64.3%).

**Figure 4:**
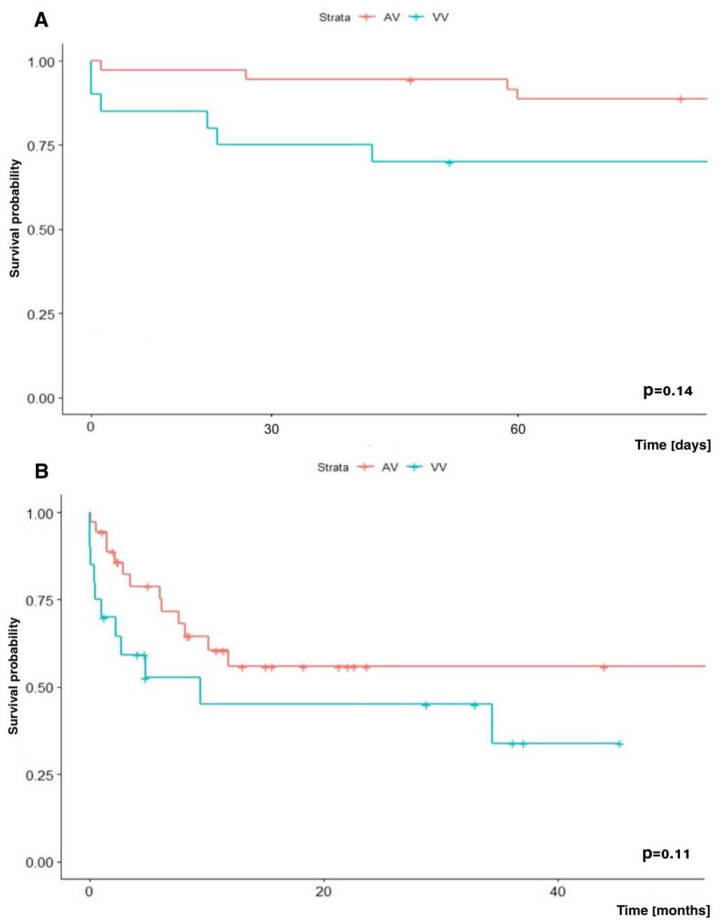
Kaplan–Meir survival analysis. (**A**) Early (60-day) survival and (**B**) long-term (two-year) survival depending on the ECMO mode: VA-ECMO vs. VV-ECMO.

In our analysis, we did not show a significant difference in early (60-day) and long-term (two-year) survival depending on the mode (VA vs. VV) in which patients were supported: 88.7% vs. 70.0% (*p* = 0.14) and 55.9% vs. 45.1% (*p* = 0.11), respectively (Figure 4).

### 3.3. Bridge to Transplantation (BTT)—Outcomes

In accordance with the latest trends in ECMO therapy, we also used it as a bridge to transplantation (BTT; *n* = 15; 26.8% of all patients on ECMO), including 12 patients (80%) who were on AWAKE-ECMO mode (RASS = 0), half of whom were extubated and half intubated (tracheostomized; not on neuromuscular blocking agents) (Figure 1; Table 4) [13]. Some of these cases of awake-ECMO that were intubated could be called an intermediate form of awake-ECMO mode, i.e., intubation performed through tracheostomy in patients treated on ECMO in awake state (Figure 1, Table 4). The median time of bridging patients with ECMO to LuTx was 19 days (Table 3).

**Figure 5:**
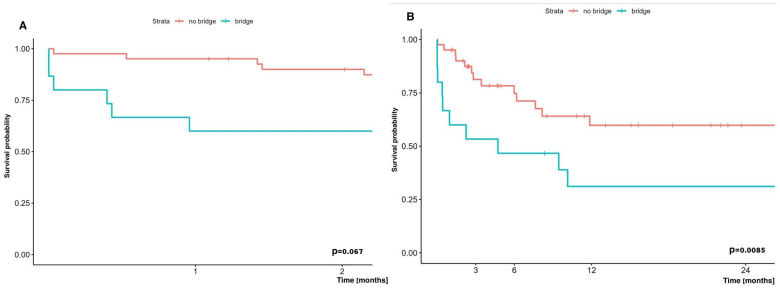
Comparison of early (60-day) (**A**) and long term (two-year) (**B**) survival of patients bridged to LuTx on ECMO vs. those who were not bridged on ECMO, but with usage of ECMO at other stages of LTx.

Taking into account the early survival (60-day) results of bridged patients to LuTx with ECMO and non-bridged with ECMO who received the ECMO system at other stages of lung transplantation, no statistically significant difference in survival was demonstrated, and was 66.7% and 90.0%, respectively (*p* = 0.067). In contrast, two-year survival of patients undergoing bridging therapy to LuTx with ECMO was significantly worse than in patients who did not undergo bridging therapy, but who received ECMO at other stages of lung transplantation, and was 31.1% vs. 59.8%, respectively (Figure 5).

### 3.4. Pulmonary Hypertension Patients and ECMO—Outcomes

**Figure 6:**
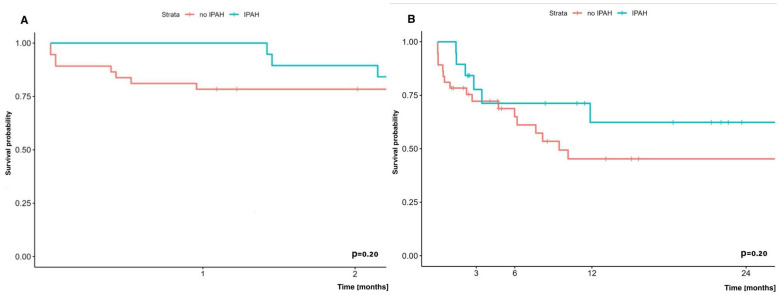
(**A**) Early (60-day) survival and (**B**) long-term (24-months) survival in patients with/without PAH in which LuTx was performed with ECMO.

Early (60-day) and long-term (two-year) survival analysis of patients with iPAH undergoing lung transplantation with ECMO did not show statistically significant differences compared to survival of patients with other diseases undergoing lung transplantation on ECMO, and was 89.5% vs. 78.4% and 62.3% vs. 45.3%, respectively (Figure 6).

### 3.5. Left Ventricle Conditioning

Achieving such results was possible thanks to applying VA-ECMO at the appropriate time and maintaining it in the postoperative period according to the concept of postoperative LV conditioning with VA-ECMO. We introduced this concept (LV conditioning) in our country in 2019, and since then, we have used it postoperatively in 13 patients (Table 4), i.e., 68% of patients with pulmonary arterial hypertension (PAH) (*n* = 13/19) and 23.2% of all ECMO population patients (*n* = 13/56). In 2022, we also successfully introduced pre-transplant LV conditioning, simultaneously conditioning injured kidneys and liver (*n* = 1; 5% of PAH patients) [9].

#### Sport ECMO (VA-SPORT ECMO and VV-SPORT ECMO)

We performed LV conditioning both in the classic VA-ECMO mode with peripheral cannulation and in six patients (*n* = 6/19; 10.7% of patients with pulmonary hypertension) in the most advanced form, i.e., in the SPORT-VA-ECMO variant, which constituted 16.7% (*n* = 6/36) of all patients undergoing lung transplantation using VA-ECMO (Table 4).

**Figure 7:**
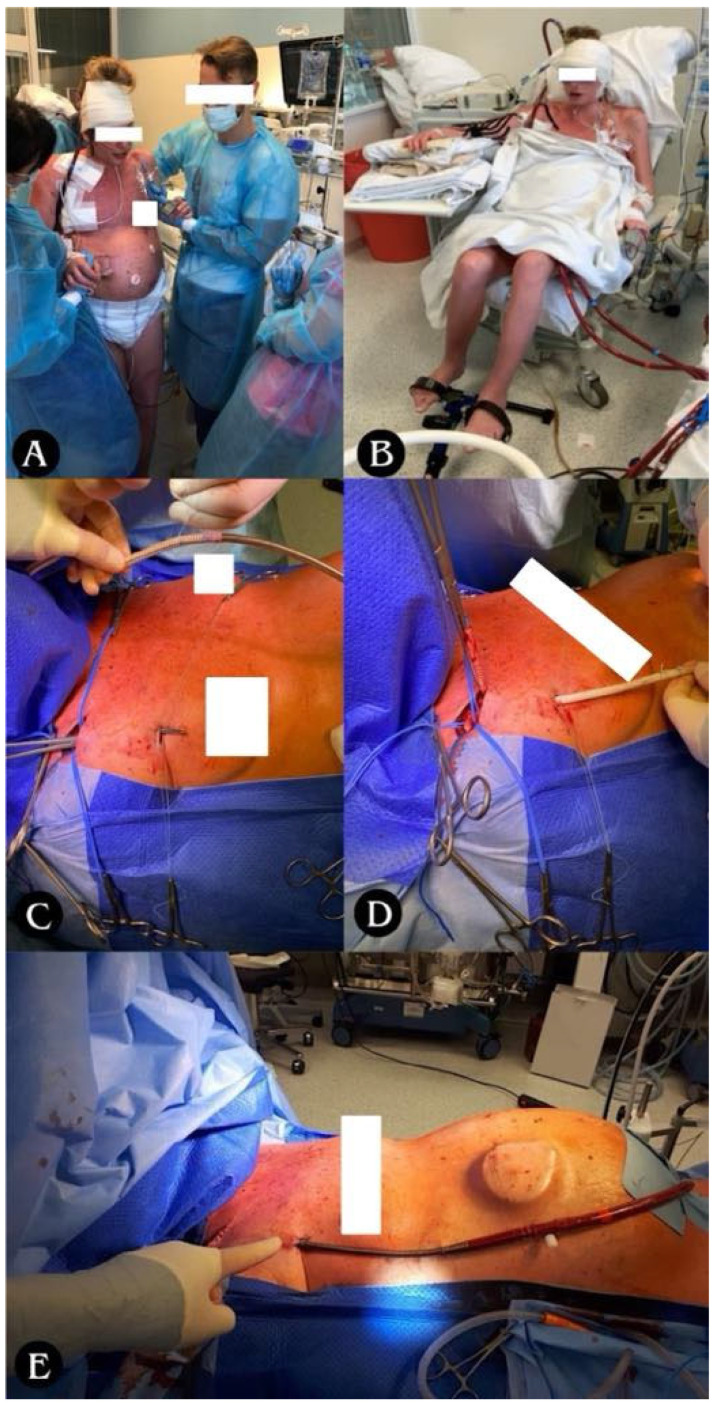
(**A**) Ability to move and (**B**) ability to rehabilitate for the duration of ECMO. (**C**–**E**) Stages of arterial cannula implantation through right subclavian/axillary artery; (**C**) tunneling of the vascular graft with arterial cannula towards the subclavicular/axillary artery; (**D**) suturing of the vascular graft to the subclavian/axillary artery with a continuous suture (non-absorbable monofilament 5/0); (**E**) closing the wound above the vascular anastomosis, attaching the cannula to the skin, starting the ECMO system.

The SPORT-VA-ECMO variant for postoperative LV conditioning assumes that the patient’s groin is free from ECMO cannulas, thanks to which the patient retains the ability to move around, fully rehabilitate, and feed orally for the duration of ECMO (Figure 7A,B). The cannulas are then inserted in the SPORT-VA-ECMO variant through internal jugular venous access (cannula drawing blood, i.e., “venous cannula”—V) and through arterial access through the right subclavian artery or right axillary artery (cannula returning blood, i.e., “arterial cannula”—A). Cannulation is associated with the risk of edema of the upper limb on the cannulation side, which was not observed in the patients operated on at our center.

**Figure 8:**
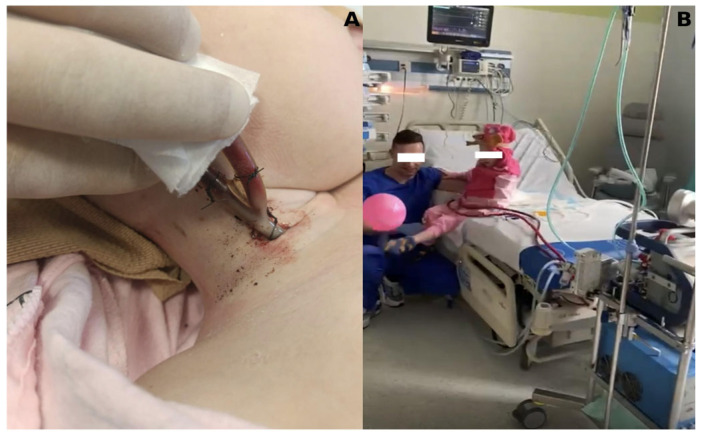
SPORT-VV-ECMO variant. (**A**) Double-lumen cannula, inserted through the internal jugular vein into the right atrium; (**B**) ability to rehabilitate for the duration of ECMO.

In the SPORT-VV-ECMO variant (used for other than PH patient support), the cannula is usually a single one, inserted through the right internal jugular vein to right atrium, and acts simultaneously as both a blood drawing and returner thanks to the special design of the cannula, called double-lumen cannula (Figure 8).

**Figure 9:**
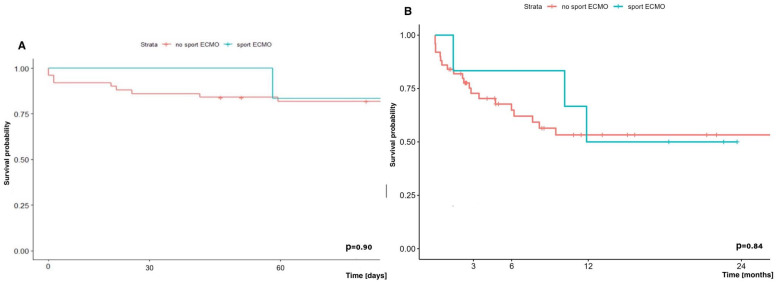
(**A**) Early (60-day) and (**B**) long-term (2-year) survival in patients supported with and without SPORT-ECMO Technique.

The results of early (60-day) survival and long-term (2-year) survival of patients supported and transplanted with SPORT ECMO did not differ significantly from the survival of patients who received other forms of ECMO support, and were 83.3% vs. 81.9% and 50.0% vs. 53.3%, respectively (Figure 9).

### 3.6. COVID-19-Related End-Stage Lung Disease; ECMO Support and Transplant—Outcomes

A new group of patients that emerged recently and attracted attention is presented in the analysis of 119 potential lung transplant candidates referred to our department during an 11-month period (from July 2020 through June 2021) following COVID-19 infection that resulted in severe respiratory failure.

**Figure 10:**
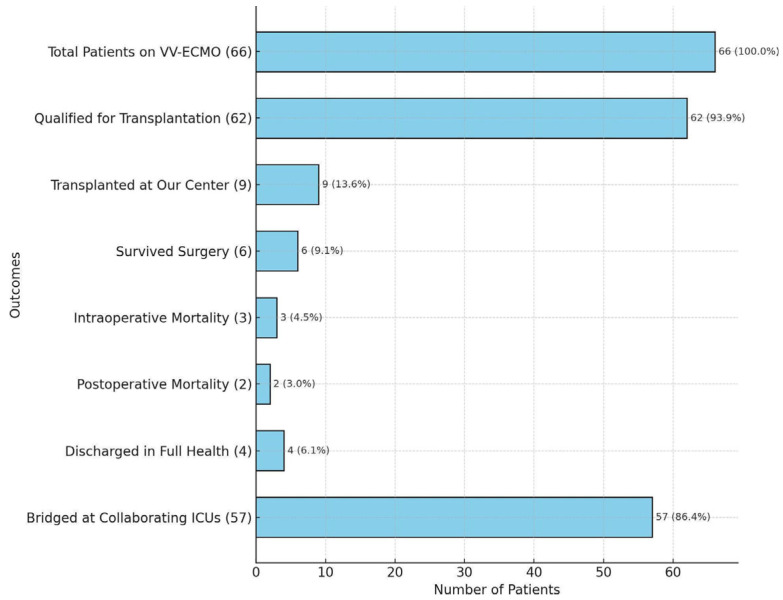
Analysis of COVID-19 patients bridged on VV-ECMO and transplanted at our center. Note: only the 9 patients transplanted at our Center were included in the analysis; the remaining patients (*n* = 57) were bridged with VV-ECMO at collaborating ICUs, but did not undergo LuTx at our center.

Among this group, all required respiratory support, including oxygen therapy, mechanical ventilation, ECMO, or a combination of ventilator and ECMO. Notably, 55.5% (*n* = 66) of these patients were placed on VV-ECMO, and the goal of the therapy was bridge to recovery, and in resistant cases not responding to treatment, bridge to lung transplantation. Out of the 119 patients, 93 (78.2%) were eligible to proceed with the lung transplant qualification process, and 62 patients (52.1%) completed the process having no contraindications. Among these 62 qualified patients, 47 (75.8%) died before a suitable organ donor could be identified, while 6 patients (9.7%) recovered sufficiently to be weaned off ECMO and mechanical ventilation and discharged home. A total of 9 out of 66 patients (13.6%) were bridged to lung transplantation using ECMO in our center, and all underwent the procedure, while the other 57 patients on VV-ECMO were managed and bridged at collaborating intensive care units, but did not proceed to transplantation at our institution (Figure 10).

When focusing on the subgroup of patients bridged on VV-ECMO at our center who ultimately underwent lung transplantation (*n* = 9), the survival rate post-surgery was 44.4% (*n* = 4/9), and the postoperative mortality rate was 22.2% (*n* = 2/9). When contextualized within the larger group of 66 patients on VV-ECMO, the survival to hospital discharge (6.1%) and postoperative mortality rate (3.0%) underscore the critical complexity of managing these cases. Outcomes reflecting significant challenges faced by this population are presented in Figure 10.

**Figure 11:**
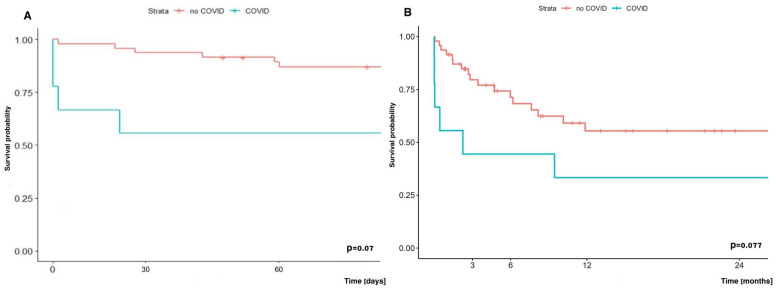
(**A**) Early survival (60-day) and (**B**) late survival (two-year) of patients with or without COVID-19 undergoing LuTx with ECMO.

Early (60-day) and long-term (two-year) survival analysis showed a trend towards worse survival in the group of COVID-19 patients supported with ECMO and undergoing LuTx vs. patients undergoing LuTx with ECMO, but without COVID-19, and it was 55.6% vs. 87.0% and 33.3% vs. 55.4%, respectively (Figure 11).

### 3.7. Heparin Usage and ECMO—Outcomes

In some cases of lung transplantation, when there was a risk of significant bleeding, e.g., coagulation disorders and hematological disorders, patients after pleurodesis, after previous interventions in the pleural cavity, specific therapy in patients with iPAH (additionally increasing the risk of hemorrhagic complications), we refrained from using heparin during ECMO therapy.

We therefore conducted an analysis of complications and survival depending on whether patients received unfractionated heparin during ECMO therapy or not. In this regard, the most worrisome complications were thromboembolic events and their most severe form, neurological complications. In our study, we did not observe a statistically significant increase in the number of such events (Table 8).

**Figure 12:**
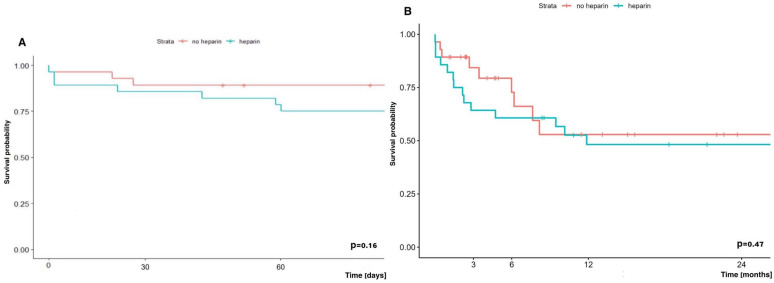
Early (**A**) and long-term (**B**) survival of patients undergoing lung transplantation with ECMO depending on heparin administration or not.

The early (60-day) and long-term (2-year) survival of patients undergoing LuTx with ECMO depending on whether they received additional doses of heparin or not, did not differ significantly, and was in the group without vs. with additional heparin at 89.3% vs. 75.0% and 52.9% vs. 48.2%, respectively (Figure 12).

## 4. Discussion

### 4.1. Introduction and Purpose of This Study

This study examines the use of ECMO in different strategies and techniques in patients with severe respiratory failure undergoing lung transplantation. Statistics show that there are more and more centers that already routinely use ECMO in almost every elective lung transplantation [14,15,16]. Based on 6 years of experience, this work contributes unique and practical insight into the evolving role of ECMO in lung transplantation, particularly in resource-constrained or developing ECMO programs. As one of the largest single-center reports from Central and Eastern Europe, this study demonstrates how various ECMO techniques—including SPORT-ECMO, awake-ECMO, and post-transplant LV conditioning—can be successfully implemented outside of high-volume Western European or North American centers. Importantly, this report illustrates real-world applicability across a broad patient spectrum, including complex cases such as retransplantations, pediatric recipients, and COVID-19-associated lung failure. While randomized data are lacking, our observational findings provide clinically relevant guidance, especially in ECMO program expansion and in centers striving to adopt more advanced ECMO protocols. The practical knowledge shared here may serve as a benchmark for other transplant centers exploring how to integrate newer ECMO strategies into their clinical workflow, while still achieving outcomes that are comparable to international benchmarks.

### 4.2. ECMO Results and Comparative Analysis

The survival results presented in this study are consistent with previously published results from other leading centers [17]. The 1-year survival rate of 85.7% and the 2-year survival rate of 51.9% in patients supported by ECMO are consistent with data from leading transplant programs worldwide. For example, the centers in Pittsburgh and Hanover reported similar 1-year survival rates, ranging from 74% to 83%, depending on the study period and patient cohort studied [14,18]. These endpoints support the role of ECMO in the treatment of critically ill patients undergoing lung transplantation, while also demonstrating the learning curve required to achieve optimal outcomes [19].

The variability in long-term survival between centers is likely related to differences in patient selection (age at transplant, disease for which the patient was transplanted, comorbidities, use or not of immunosuppression), ECMO protocols, and postoperative management (type and level of immunosuppression in the long term). For example, early survival rates in the initial Pittsburgh studies were lower, likely due to earlier stages of ECMO program development [18]. Over time, steady improvements have shown that ECMO can be successfully incorporated into the lung transplant process, provided that the approach is tailored to the strengths of the centers and patient characteristics.

### 4.3. VA vs. VV ECMO

Patients requiring VA-ECMO support usually have higher risk of complications and death, which is associated with limb ischemia distal to the area where cannulation is performed, occurrence of Harlequin syndrome, and because these patients necessitating VA-ECMO are more likely to have preoperative circulatory failure, cardiac dysfunction, or pulmonary hypertension, which obligate infusion of catecholamines with its widely known side effects [20,21,22].

VA-ECMO is often used in patients who present with more severe diseases described above, while VV-ECMO is reserved for pure respiratory failure. In our center, the dominant form of ECMO therapy was VA (*n* = 36/56; 64.3%) vs. VV (*n* = 20/56; 35.7%). VA-ECMO is often considered more demanding to manage, with more complications (such as vascular complications due to the need for arterial cannulation or thromboembolic complications), and therefore it is believed that the results in terms of early and long-term survival should also be worse compared with VV-ECMO. However, meticulous perioperative care and advanced protocols have mitigated the differences in outcomes, while increasing the flexibility of ECMO to meet different clinical needs without compromising long-term survival [2,23,24,25,26,27,28,29,30,31]. Comparative analyses of VA versus VV ECMO suggest that while VV is associated with fewer vascular and bleeding complications, outcomes are heavily influenced by patient selection and indication for support [18]. This comparison proved that there was no statistically significant difference in survival outcomes between VA and VV ECMO modes. One-year survival was 88.7% for VA-ECMO and 70.0% for VV-ECMO, with two-year survival rates of 55.9% and 45.1%, respectively. Although VV-ECMO is generally considered to be less risky than VA-ECMO, our VV-ECMO cohort included a disproportionately large number of critically ill COVID-19 survivors who required ECMO as a bridge to transplant. These patients had significantly longer ECMO duration, more frequent mechanical ventilation, and impaired renal function. This subgroup composition likely contributed to the relatively poorer survival outcomes observed in the VV group and should be considered when interpreting ECMO-related mortality in this context.

These results are consistent with previously reported findings by other research groups that suggest that ECMO modality, regardless of whether it primarily supports cardiac or respiratory function, does not inherently affect long-term survival when managed appropriately. This observation is consistent with the long-term data presented by the Hannover group, which showed no significant difference in post-transplant mortality depending on the type of ECMO, but rather on patient characteristics [14].

### 4.4. Awake ECMO and Rehabilitation

Awake ECMO is an innovative method that has been developed over the last few years and is gaining popularity, as many reports have shown that it not only reduces the number of complications related to prolonged sedation and intubation, but also improves survival at 6 months of follow-up. There is significant evidence from studies that have shown other benefits of awake ECMO in selected patients undergoing bridging to lung transplantation, enabling spontaneous breathing and early mobilization without the need for mechanical ventilation [32]. In this study, awake ECMO was used in completely extubated patients (*n* = 6) as well as in tracheostomized patients (*n* = 6) who were continuously or intermittently supported by mechanical ventilation, which allowed for mobilization and preoperative improvement of their condition. All of these awake patients who were planned for transplantation ultimately underwent transplantation, but we did not analyze the efficacy of rehabilitation between these subgroups (sedative vs. awake extubated vs. tracheotomized). However, such an analysis would be extremely interesting and informative, and will certainly be performed once a larger number of patients are enrolled.

Reports from other institutions suggest benefits, emphasizing the potential of awake ECMO to improve survival by, among other things, preserving diaphragmatic function and reducing postoperative complications [33].

### 4.5. SPORT-ECMO

In addition, we have also used advanced ECMO configurations such as SPORT-ECMO, allowing patients to remain conscious and fully mobile while on ECMO support. The “Sport ECMO” technique using subclavian artery cannulation in the case of VA-ECMO or cannulation of the superior vena cava via the internal jugular vein using double-lumen cannula (Avalon) in the case of VV-ECMO allows for greater patient mobility and participation in physiotherapy during the use of appropriate ECMO support techniques (VV or VA) [34].

This technique (subclavian access) has been extensively and thoroughly described (including surgical description) by the Columbia University group, although not in lung transplantation surgery [34,35,36]. It utilizes subclavicular/axillary cannulation, leaving the femoral vessels free, allowing patients to stand upright, move around, and enhance rehabilitation efforts, while minimizing risks such as limb ischemia, which may be increased in non-sport ECMO techniques, where appropriate limb protection is required by implantation of a nutrient cannula [37]. Although complications such as upper limb edema or infections have been reported in other studies, they were successfully treated in our cohort, confirming the feasibility of this advanced strategy [38]. In the presented study, in the group of patients with sport ECMO, we did not observe a typical complication such as upper limb edema, although complications with the cannulation site in VA-ECMO were noted in three cases, which constituted 5.4% of the entire studied population of patients undergoing lung transplantation with the use of ECMO (Table 5).

### 4.6. Bridge to Lung Transplantation (BTT)

Patients undergoing bridge to lung transplantation using ECMO are at increased risk for a variety of complications (vascular complications related to cannulation, hemorrhagic, thromboembolic, infectious), with this study showing a 2-year survival of 31.1% compared with 59.8% in patients receiving intraoperative or postoperative ECMO alone.

In addition, a more frequent occurrence of comorbidities was observed in the group of patients bridged to lung transplantation with ECMO, as well as a more frequent need for mechanical ventilation before ECMO initiation and a longer ECMO duration until transplantation. Especially, the longer ECMO duration in this group may serve as a surrogate marker of disease severity before transplantation. It can therefore be assumed that these patients constitute a particularly high-risk population, which may explain their lower long-term survival despite similar early post-transplant outcomes, although these results should be interpreted with caution due to the small sample size and retrospective nature of our study.

The number of bridges to transplantation (BTT) was 15, which is 26.8% of all ECMO runs performed in our center and, compared to other leading centers, this is a quite large number. In the material from the center in Hannover, out of 917 patients undergoing LuTx, 68 (i.e., 7%) required the use of ECMO as a bridge to transplantation. Their study found hospital mortality to be significantly higher in patients who required pre-transplant ECMO bridging therapy compared with those who did not require such therapy (15% vs. 5%, *p* = 0.003) [14]. The survival of patients bridged to ECMO to transplantation and undergoing transplantation at the center represented by Hozenaker was 66% (1 year), 58% (3 years), and 48% (5 years), whereas among patients bridged to retransplantation surgery, it was significantly shorter, and the median survival in this group was 15 months [10].

This disparity highlights the challenges faced by patients requiring long-term ECMO support prior to transplantation (as a bridge to transplant), who often experience exacerbations of underlying disease, secondary organ failure, or severe pulmonary hypertension. Despite these challenges, BTT remains a key strategy in saving patients who have no other therapeutic options, offering a chance of survival in scenarios that would otherwise be fatal [39]. The literature comparing ECMO bridge with mechanical ventilation indicates that ECMO provides better survival before and after transplantation, probably due to less lung damage to the patient awaiting LTx, better gas exchange, and preservation of diaphragmatic function, which will be critically needed for proper rehabilitation immediately after LuTx [39].

One of the reasons for the worse results presented in comparison to other centers could be the fact that among the patients from our study group, there were COVID-19 patients, which at that time was a new disease entity with high mortality. Our work is one of the first to present the results of ECMO in patients who underwent lung transplantation after COVID-19, and the presented results may reflect this fact. A detailed analysis of the worse survival will be performed after collecting more data.

### 4.7. Pulmonary Arterial Hypertension (PAH) and ECMO

PH is the result of such devastating diseases as idiopathic pulmonary fibrosis (IPF) or pulmonary arterial hypertension (PAH), including the idiopathic form of PAH (iPAH). Patients with idiopathic pulmonary arterial hypertension (iPAH) undergoing lung transplantation are among the most difficult to treat perioperatively due to severe right ventricular dysfunction and, as reported by many scientific teams, potential postoperative LV failure [40,41,42,43]. This LV failure is associated with the fact that for the duration of the disease (sometimes even several years), the left ventricle undergoes fibrosis, interestingly due to the low workload/load—(‘preload’)—due to impaired blood flow through the pulmonary vascular bed in the course of pulmonary hypertension [42,43,44]. Lung transplantation with an already low-resistance vascular bed causes a dramatic increase in left ventricular preload, which has been “accustomed” to low preload for many years. Early survival in this study was 89.5%, and two-year survival was 62.3%, comparable to patients without PAH.

These results were achieved thanks to advanced protocols, including postoperative LV conditioning, which gradually adapts the left ventricle to the increased preload after transplantation [45].

Other studies have also highlighted similar challenges in the treatment of patients with iPAH and reported similar outcomes [10]. The use of VA-ECMO for several days as left ventricular conditioning in these cohorts was effective in mitigating the above-described risks, further supporting its role in the treatment of this high-risk group of patients.

### 4.8. COVID-19 and Lung Transplantation with ECMO

The COVID-19 pandemic has become an event that has posed new challenges to medicine. It quickly became clear that lung transplantation is the only and final way to save patients with severe respiratory failure whose lungs have been completely destroyed, while ECMO has often been the only real bridge to recovery or to transplantation for patients with severe ARDS refractory to conventional treatment [46,47,48].

The emergence of end-stage lung injury associated with COVID-19 has contributed significantly to the increase in the number of patients requiring ECMO as a bridge to transplantation. These patients were exclusively supported with VV-ECMO for isolated respiratory failure (Figure 8), in contrast to patients with iPAH, who more frequently required VA-ECMO. This change in the clinical phenotype of patients and ECMO configuration underlies the increasing trend in ECMO use during the pandemic years.

In our study, early survival in patients affected by COVID-19 after lung transplantation with ECMO was 55.6%, compared to 87.0% in patients undergoing lung transplantation with ECMO, but not affected by COVID-19. This discrepancy, in our opinion, reflects the cumulative impact of prolonged critical illness, including multiple organ dysfunction commonly observed in patients with COVID-19. Other centers have reported similar results. For example, early data from centers performing lung transplants in COVID-19 patients showed that survival was poorer in patients with additional comorbidities, such as renal or hepatic dysfunction, compared with patients with isolated respiratory failure.

Better outcomes after lung transplantation were achieved in centers that had a policy of transplanting patients with “single organ failure”—in this case, only lung failure [49,50,51]. This underscores the importance of careful patient selection and advanced perioperative management strategies. These and other challenges underscore the need for further refinement of transplant procedures and ECMO protocols for this unique patient population.

### 4.9. Anticoagulation Protocols

Anticoagulation during ECMO therapy requires a careful approach and a balance between thromboembolic and hemorrhagic risk. Recent advances in ECMO design, including heparin-coated cannulas and low-resistance oxygenators, have facilitated the use of heparin-free protocols in high-risk populations such as patients with prior pleurodesis or bleeding tendencies.

In this study, there was no statistically significant difference in survival, thromboembolic, or hemorrhagic complications between patients treated with or without therapeutic doses of heparin. All patients undergoing ECMO therapy had their coagulation status assessed pre- or post-transplant using APTT and intraoperatively using ACT, and the recommended anticoagulation levels were in the range of 50–60 s and 180–220 s, respectively.

Our results are consistent with reports from other centers that suggest that individualized anticoagulation protocols can reduce the complications mentioned above without compromising outcomes [52,53,54]. This approach is particularly important in patients receiving long-term ECMO support (sometimes for many weeks), who are at increased risk for bleeding and thrombotic events.

### 4.10. Limitations and Future Directions

Our study provides insights into the research area, although its retrospective nature and the heterogeneity of the patient population analyzed make some limitations. For example, small subgroup sizes in advanced ECMO techniques such as SPORT-ECMO limit the statistical power of the analyses, and from a statistical point of view, one should be careful with these results and especially with their application in daily practice. The authors will certainly undertake further analyses after increasing the number of patients treated with ECMO and lung transplantation. The prospective and randomized studies that are already underway will certainly significantly enrich the knowledge on the use of ECMO in lung transplantation.

Future studies should focus on multicenter collaborations to validate these findings and establish standardized ECMO protocols. Additionally, future studies should include the population of pediatric patients in terms of slightly different circulatory and respiratory physiology.

Moreover, new areas of exploration should include the development of hybrid ECMO configurations, miniaturized systems, and extension indications for ECMO in high-risk transplant populations. Continued innovation in perioperative care and multidisciplinary approaches will be essential to further improve lung transplantation outcomes.

## 5. Conclusions

This study confirms the established role of ECMO in modern lung transplantation, demonstrating its versatility in addressing a variety of clinical scenarios, including PAH and COVID-19. This study indicates that despite the challenges associated with high-risk populations, ECMO enables successful transplantation and survival outcomes comparable to data from leading centers. With continued innovation in ECMO technology and protocols, its potential in lung transplantation will continue to expand, providing hope to the sickest patients.

## Figures and Tables

**Table 1:** Types and frequency of diseases due to which patients underwent lung transplantation using the ECMO system.

Disease	*n*	%
iPAH	19	33.9
COVID-19	9	16.1
Idiopathic Pulmonary Fibrosis	8	14.3
Cystic Fibrosis	4	7.1
Retransplantation	4	7.1
Rescue	4	7.1
CTEPH	1	1.8
COPD	1	1.8
Emphysema	1	1.8
Silicosis	1	1.8
Langerhans cell histiocytosis	1	1.8
Sarcoidosis	1	1.8
Mounier–Kuhn Syndrome	1	1.8
Rendu–Osler–Weber Syndrome	1	1.8
Total	56	100

COPD—chronic obstructive pulmonary disease; CTEPH—chronic thromboembolic pulmonary hypertension; iPAH—idiopathic pulmonary artery hypertension.

**Table 2:** Demography, characteristics of the patients, and comorbidities.

Variable	*n*	%						
Female	27	48.2						
Men	29	51.8						
Hypertension	6	10.9						
Osteoporosis	2	3.6						
Renal insufficiency	4	7.1						
Diabetes	3	5.4						
Mechanical Ventilation before ECMO	7	16.3						
Neurological complications before ECMO	3	5.4						
**Variable**	***n***	**min**	**max**	**Me**	**Q1**	**Q3**	**x**	**SD**
Age [years]	56	18.00	65.00	40.00	31.5	51.0	37.6	13.4
Body mass [kg]	50	20.5	102.0	62.2	54.0	73.5	63.4	17.1
Height [cm]	50	130.0	197.0	168.0	162.0	176.0	169.0	10.7
BMI [kg/m^2^]	49	12.0	33.2	21.8	19.5	24.9	21.9	4.67

*n*—number, min—minimum, max—maximum, Me—median, Q1—first quartile, Q3—third quartile x—mean, SD—standard deviation.

**Table 3:** Characteristics of patients’ ECMO runs.

Variable	*n*	min	max	Me	Q1	Q3	x	SD
ECMO total time [hours]	56	2.0	2166.0	66.2	7.05	266.0	232.0	398.0
Hospitalizationtime after decannulation [days]	55	0.0	140.0	29.0	21.5	41.5	34.0	26.6
Bridge to transplant time [days]	15	2.0	90.0	19.0	15.5	28.0	24.7	20.8
Left ventricle conditioning time [days]	13	0.0	45.0	4.0	3.0	7.0	10.3	15.1

*n*—number, min—minimum, max—maximum, Me—median, Q1—first quartile, Q3—third quartile x—mean, SD—standard deviation.

**Table 4:** The total number of ECMO performed in the years 2018–2023, divided into the number of bridges of patients to LuTx (bridge to LuTx) and left ventricular conditioning (LV conditioning), and the number of ECMO-awake (RASS = 0; alert and calm), divided into intubated and extubated and sport ECMO.

Procedure	2018	2019	2020	2021	2022	2023	Total	Total [%] *
Bridge to LuTx	2	3	3	6	0	1	15	6.84
AWAKE ECMO	2	3	3	2	2	0	12	5.47
LV Conditioning with ECMO	0	3	1	3	5	1	13	5.93
SPORT ECMO	1	0	1	2	2	0	6	3.73
iPAH with VA-ECMO	0	4	1	5	4	5	19	8.67
COVID-19 with VV-ECMO	0	0	3	6	0	0	9	4.10
LuTx with ECMO	5	6	7	12	14	12	56	25.57
Total LuTx	27	36	29	41	50	36	219	-
ECMO AWAKE Mode								
AWAKE-INTUBATED ECMO (tracheotomized)	1	2	1	1	1	0	6	2.73
AWAKE-EXTUBATED ECMO	1	1	2	1	1	0	6	2.73

iPAH—idiopathic pulmonary arterial hypertension. * Percentage from total LuTx.

**Table 5:** Comparison of statistically significant outcomes in patients undergoing BTT and non-BTT patients.

Variable	BTT Patients *n* = 15	Non-BTT Patients *n* = 41	*p*
Median ECMO duration [hours]	456.00	29.50	0.00
Mechanical ventilation prior ECMO	6	1	0.00
Renal insufficiency prior to ECMO	3	1	0.03
Neurological complications prior to ECMO	2	1	0.10

**Table 6:** Comparison of statistically significant outcomes in patients VA and VV ECMO.

Value	VA * n = 36	VV *n* = 20	*p*
Demography			
Age [years]	41.00	36.50	0.05
RVSP [mmHg]	80.00	34.00	0.00
Comorbidities			
Pulmonary hypertension	33	1	0.00
iPAH	19	0	0.00
COVID-19	0	9	0.00
Procedure			
Bridge to LuTx	2	13	0.00
Characteristics of patients’ ECMO runs			
ECMO total time [hours]	9.25	293.00	0.01
Bridge to transplant time [days]	2.00	13.00	0.00

* VA + VAV.

**Table 7:** Mortality rate and frequency of complications in patients undergoing lung transplantation using the ECMO system.

Variable	*n*	%
Death during ECMO run	10	17.90
30-day mortality	7	12.50
Cannulation site complications	3	5.40
Neurological complications	5	8.90
Hemorrhagic complications	12	21.40

**Table 8:** Type and frequency of complications depending on the use or not of additional doses of heparin.

	Additional Heparin After ECMO Initiation				
	No		Yes		
	*n*	%	*n*	%	*p*
Sex (male)	15	53.6	14	50.0	0.999
Neurological complications	2	7.1	3	10.7	0.999
Cardiological complications	5	17.9	4	14.3	0.999
Hemorrhagic complications	4	14.3	8	28.6	0.329

## Data Availability

The anonymized dataset supporting the findings of this study is openly available upon reasonable request to the corresponding author. In accordance with institutional and ethical guidelines, the dataset has been deidentified to ensure patient confidentiality. We fully support the principles of Open Data and transparency in scientific research. Therefore, researchers interested in replicating or expanding upon our analyses are welcome to request access to the data, which will be shared in compliance with ethical regulations and applicable data use agreements. For access to the dataset, please contact Dr. Tomasz Stącel at tstacel@tlen.pl.

## Abbreviations

The following abbreviations are used in this manuscript:

ARDS	acute respiratory distress syndrome
BTT	bridge to transplantation
CARDS	coronavirus acute respiratory distress syndrome
CFV	common femoral vein
CFA	common femoral artery
CLAD	chronic lung allograft dysfunction
COPD	chronic obstructive pulmonary disease
CTEPH	chronic thromboembolic pulmonary hypertension
ECMO	extracorporeal membrane oxygenation
FA	femoral artery
IJV	internal jugular vein
iPAH	idiopathic pulmonary hypertension
IPF	idiopathic fibrosis
IQR	inter-quartile range
LuTx	lung transplantation
LV	left ventricle
PAH	pulmonary artery hypertension
PGD	primary graft dysfunction
PH	pulmonary hypertension
RV	right ventricle
SD	standard deviation
A	veno-arterial
VAV	veno-arterio-venous
VV	veno-venous
